# Effects of warm compress on tear film, blink pattern and Meibomian gland function in dry eyes after corneal refractive surgery

**DOI:** 10.1186/s12886-021-02091-2

**Published:** 2021-09-10

**Authors:** Xueyi Zhou, Yang Shen, Jianmin Shang, Xingtao Zhou

**Affiliations:** 1 Department of Ophthalmology and Optometry, Eye and ENT Hospital of Fudan University, Shanghai, China; 2 China Eye Institute and Department of Ophthalmology, Eye and ENT Hospital of Fudan University, No.19 Baoqing Road, 200031 Shanghai, China; 3 NHC Key Laboratory of Myopia (Fudan University), Key Laboratory of Myopia, Chinese Academy of Medical Sciences, 200031 Shanghai, China; 4 Shanghai Research Center of Ophthalmology and Optometry, Shanghai, China; 5 Shanghai Engineering Research Center of Laser and Autostereoscopic 3D for Vision Care (20DZ2255000), Shanghai, China

**Keywords:** Dry eye, SMILE, LASEK, Warm compress

## Abstract

**Background:**

To assess the effects of warm compress (WC) on tear film lipid layer, blink pattern and Meibomian gland function in patients with dry eye following femtosecond laser small incision lenticule extraction (SMILE) and laser-assisted subepithelial keratomileusis (LASEK).

**Methods:**

We enrolled 37 eyes of 37 participants, each with dry eye for more than 2 years following SMILE (25 eyes) or LASEK (12 eyes). WC was performed using a spontaneously heating eye mask. Tear film break-up time (TBUT), tear film lipid layer thickness (TFLLT), blink pattern, Meibomian secretory function scores (MGS), visual acuity, spherical equivalent (SE), keratometry, central corneal thickness (CCT) and aberration were assessed before and after WC.

**Results:**

After WC, the following mean values all increased relative to baselines: CCT, SE, minimum (Min-), maximum (Max-) and average (Ave-) TFLLT, TBUT, total MGS (TMGS), number of glands secreting any liquid (MGL), and complete blink rate (CBR) (*p* values ranging from < 0.001 to 0.042). Partial blink frequency (PBF) and partial blink rate (PBR) decreased (*p* = 0.002 in both cases). The decrease of PBF was higher in SMILE subgroup than in LASEK (*p* = 0.030). TBUT variation was positively correlated with that of Ave-TFLLT and TMGS (*p* = 0.046, 0.028, respectively). Max-TFLLT variation was correlated with that of TMGS (*p* = 0.020).

**Conclusions:**

WC may temporarily increase tear film thickness and stability, decrease partial blink, and partly augment Meibomian gland function in dry eye patients after corneal refractive surgeries. Future studies are required to investigate long term clinical efficacy and safety.

## Background

With the incidence of myopia increasing globally [1], keratorefractive procedures have gained widespread popularity in recent decades. With the benefit of novel techniques, especially the application of femtosecond laser in ophthalmology, keratorefractive procedures are now less invasive and safer, and correct refractive error more accurately than in the past.

Small incision lenticule extraction (SMILE) is the latest keratorefractive procedure. A refractive stromal lenticule is created using a femtosecond laser and extracted through a small peripheral incision, thus modifying the corneal shape and correcting refractive errors [2]. Laser-assisted subepithelial keratomileusis (LASEK) is a surface ablation procedure, which creates an epithelial flap only on the cornea. As both SMILE and LASEK avoid stromal flap creation, fewer corneal nerve fibers are severed, with concomitantly faster regeneration of the corneal nerves and recovery of corneal sensation relative to traditional laser-assisted in situ keratomileusis (LASIK) or femtosecond laser assisted LASIK (FS-LASIK) [3–5].

Nevertheless, as neural damage cannot be avoided completely, post-operative dry eye is still a common complication following SMILE and LASEK. Warm compress (WC) is effective for relieving dry eye syndrome or Meibomian gland dysfunction (MGD) [6, 7]. Here, a novel instrument was employed for assessment of dry eyes to determine the effects of WC on tear film thickness and Meibomian gland function as accurately as possible in post-operative patients.

## Methods

### Patients

 Participants were recruited between July 2017 and October 2017 at the Department of Ophthalmology, Eye and ENT Hospital of Fudan University (Shanghai, China). The study adhered to the tenets of the Declaration of Helsinki and was approved by the Ethical Committee of the Fudan University Eye and ENT Hospital Review Board. All participants were fully informed and gave written consent for publication of this information.

Sample size was calculated by Power and Sample Size Calculators (http://powerandsamplesize.com/Calculators/). Using the data of Tan J, et al. measured by Lipiview [8] and calculation method recommended in the Cochrane Handbook (SD_change_=[SD^2^_baseline_ + SD^2^_final_-2 x Corr x SD_baseline_ x SD_final_]^1/2^, the correlation between baseline and endpoint was set to 0.5) [9, 10], a sample size of 14 participants was required to detect a treatment effect in TFLLT of 16.90 nm, with a standard deviation (SD) of 22.06 nm, with 80 % power at the 5 % level of statistical significance. Thirty-seven patients were enrolled. Among them, 25 underwent SMILE, and the remaining 12 underwent LASEK. Inclusion criteria were as follows: age ≥ 18 years, reported dry eye syndromes for more than 2 years following refractive surgeries with a tear film break-up time (TBUT) < 10 s. Before refractive surgery, all patients have no complain of dry eye syndrome and the TBUT was longer than 10 s. Exclusion criteria included: the use of topical medications (except ocular lubricants or artificial tears), other dry eye or MGD related therapy at least 1 month prior to the study; active eye inflammation; experienced post-surgical severe ocular or systemic diseases or other long-term complications except dry eye. All patients were instructed to stop using topical medications or wearing contact lenses at least 24 h before examinations, and to avoid skin care products or make-up around the eyes. All patients were routinely screened preoperatively and met the criteria for SMILE or LASEK, and all surgeries went smoothly and were performed by the same experienced surgeon (XTZ).

### Surgical procedures

We used the VisuMax femtosecond laser system (Carl Zeiss Meditec, Jena, Germany) to perform SMILE and the Mel-80 excimer laser system (Carl Zeiss Meditec AG, Jena, Germany) to perform LASEK. We previously described these procedures in detail [11, 12].The femtosecond laser settings were as follows: 500 kHz repetition rate, 130 nJ pulse energy, 110 to 120 μm intended cap thickness, 6 to 6.5 mm optical zone, 7.3 to 7.5 mm cap diameter, and a 2 mm side cut at the 12 o’clock position.

### Clinical examinations

Study outcome parameters were assessed in the same order before and at 5 min after WC, from least to most invasive: corneal curvature (K1, K2, Km) and central corneal thickness (CCT) measured by Pentacam HR (Oculus GmbH, Wetzlar, Germany), uncorrected distance visual acuity (UDVA), corrected distance visual acuity (CDVA) and spherical equivalent (SE), tear film lipid layer thickness (TFLLT), blink frequency, TBUT, and Meibomian secretary function scores. All examinations were performed between 11:00 am and 17:00 pm in the same room by a single pre-trained and experienced technician. The indoor temperature was set to 22–25 °C.

TBUT was measured using a single fluorescein strip (Jingming New Technology Development Co., Ltd, Tianjin, CHN) to the inferior palpebral conjunctiva after moistening by a drop of normal saline. Time was recorded by digital clock. TBUT was measured three times and the mean was calculated and used in the statistical analyses.

### Warm compress

All patients were treated with a warm compress using a spontaneous heating eye mask (Zhenshiming Pharmaceutical Co., Ltd, Fuzhou, Jiangxi, CHN) for 20 min in both eyes according to the manufacturer’s instruction. This eye mask is a standardized, fragrance free, over-the-counter eyeshade, certified after examination and verification by Shanghai Institute of Quality Inspection and Technical Research. At an ambient temperature of 25 ± 2 °C, it takes an average of 3 min to warm up to 35 °C, and maintains temperature over 35 °C for an average of 30 min. The mean temperature was 40.7 °C.

### LipiView

An interferometer (LipiView, TearScience® Inc, Morrisville, NC) was used for measuring the quantitative TFLLT and blink frequency dynamically by analyzing more than one million tear film data points. TFLLT was calculated using interferometry color described as interferometric color units (ICUs), which is equivalent to 1 nanometer (nm). Patients were instructed to hold their head in a comfortable position and look directly into the camera without deviation of eye position, and to blink freely throughout imaging. Natural light from the source passed through the tear film and was reflected back to the camera. The measurement region was the lower third of the cornea, approximately 1 mm above the inferior tear meniscus. The minimum (Min-), maximum (Max-), average (Ave-) and standard deviation (-std) TFLLT were analyzed automatically within preset 19.1 s. Simultaneously, total (TBF) and partial blink frequency (PBF) were also recorded, and were used to calculate partial blink rate (PBR, calculated by PBF/TBF) and complete blink rate (CBR, calculated by 1-PBR). The upper cut-off of LipiView is 100 ICU; values higher than this are recorded as 100 + ICU. A conformance factor for imaging ≥ 0.7 was considered acceptable, but to ensure quality of our data, only conformance factor ≥ 0.8 were included in the final analysis.

### Meibomian gland evaluator

Secretory function of the Meibomian glands was measured by a handheld Meibomian Gland Evaluator (TearScience® Inc, Morrisville, NC) and recorded as Meibomian gland scores. Gentle and blink-stimulated pressure (0.8 g/mm^2^ − 1.2 g/mm^2^) was applied to the Meibomian gland openings along the lower eyelid margin. The width of the evaluator covers an average of 5 gland openings. After gentle clean of the lower eyelid using clean cotton swabs, the evaluator was applied 1–2 mm below and in parallel with the eyelid, near the root of the eyelash. The eyelid was gently turned inside out slightly just until the gland opening was clearly visible. Fifteen glands in 3 regions (temporal, central, and nasal) were evaluated, with an average pressing time of 10–15 s. We graded the number and secretion characteristics of glands: 3 points (clear liquid), 2 points (cloudy liquid), 1 point (toothpaste-like), and 0 (no secretion). The following metrics were calculated: the total Meibomian gland secretion score (TMGS) of all 15 glands, ranging from 0 to 45; the number of glands secreting any liquid (MGL, clear or cloudy liquid, grade 2 or 3); and the number of glands secreting clear liquid (MGC, clear liquid, grade 3).

### Statistical analysis

Statistical analysis was performed using SPSS ver. 22.0 (SPSS Inc, Chicago, IL, USA). The right eye was selected as the study eye. Variables were described as averages ± SD. The one-sample Kolmogorov-Smirnov test was used to test for normality. Paired t-tests or Wilcoxon tests were then used to analyze the changes from baseline in normal or non-normal distribution parameters, respectively. Variables were compared between SMILE and LASEK using independent sample t-tests or Mann-Whitney U tests. Pearson or Spearman correlation analyses were used to evaluate the linear relationships between different variables. *P* < 0.05 was considered statistically significant.

## Results

 We enrolled 37 right eyes of 37 participants (age 29.5 ± 6.3 years, 14 male and 23 female). The mean time interval since refractive surgery was 29.10 ± 2.70 months (range from 23 to 33 months). Before WC, there was no difference in all indicators between surgeries.

### TFLLT

**Fig. 1:**
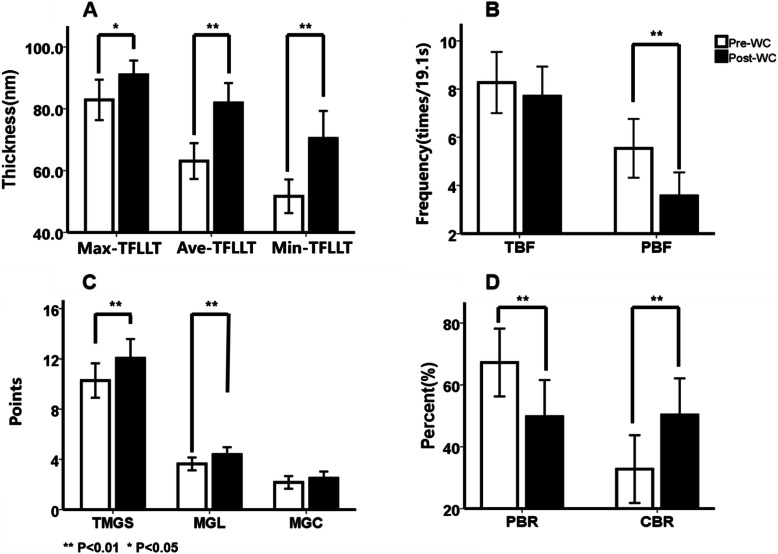
Change in TFLLT, blink frequency, meibomian glands scores and blink rate after WC. (**A**: Min-, Max- and Ave-TFLLT; **B**: total and partial blink frequency; **C**: different meibomian glands scores; **D**: complete and partial blink rate)

**Fig. 2:**
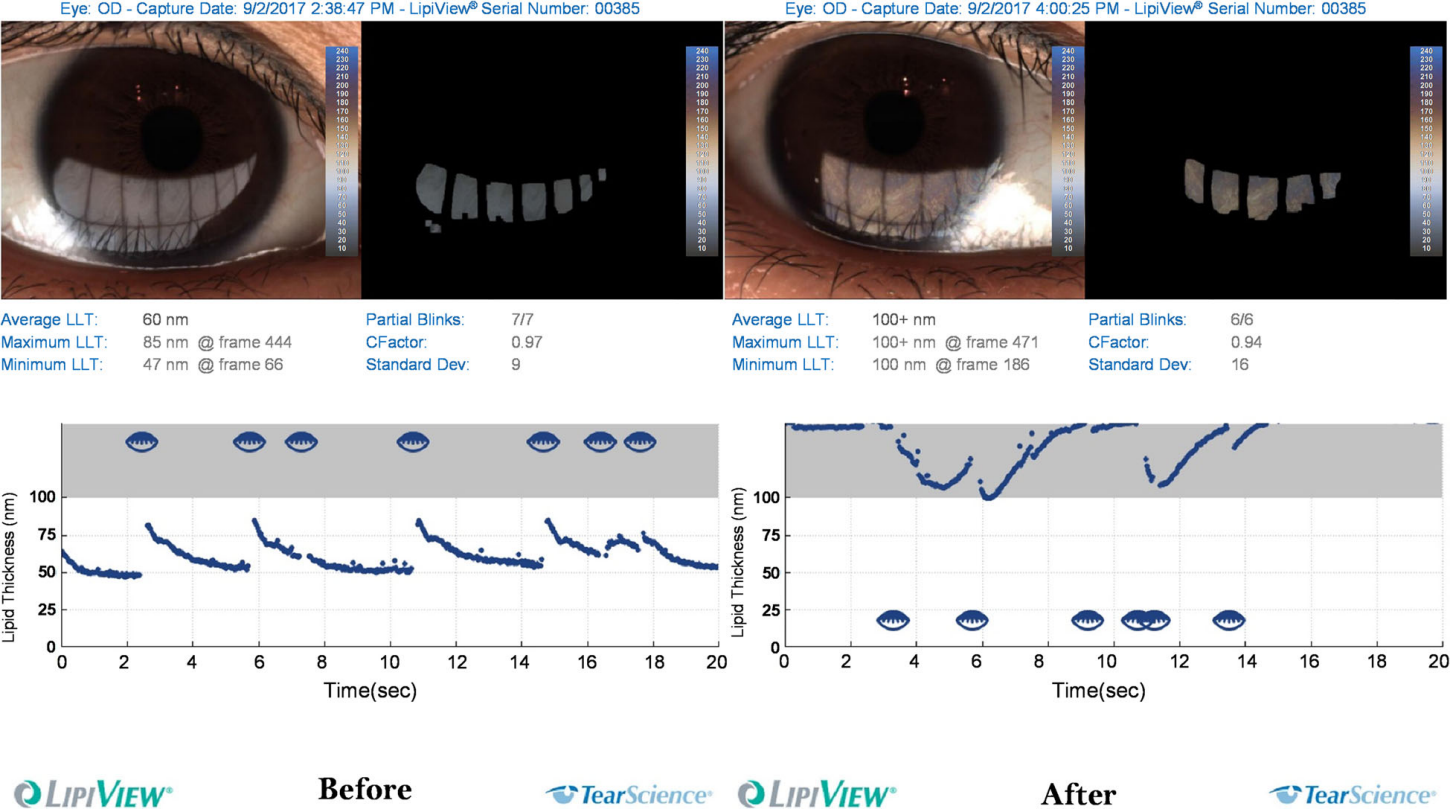
Interference pattern of a patient before (**A**) and after (**B**) warm compress under LipiView inspection window

**Fig. 3:**
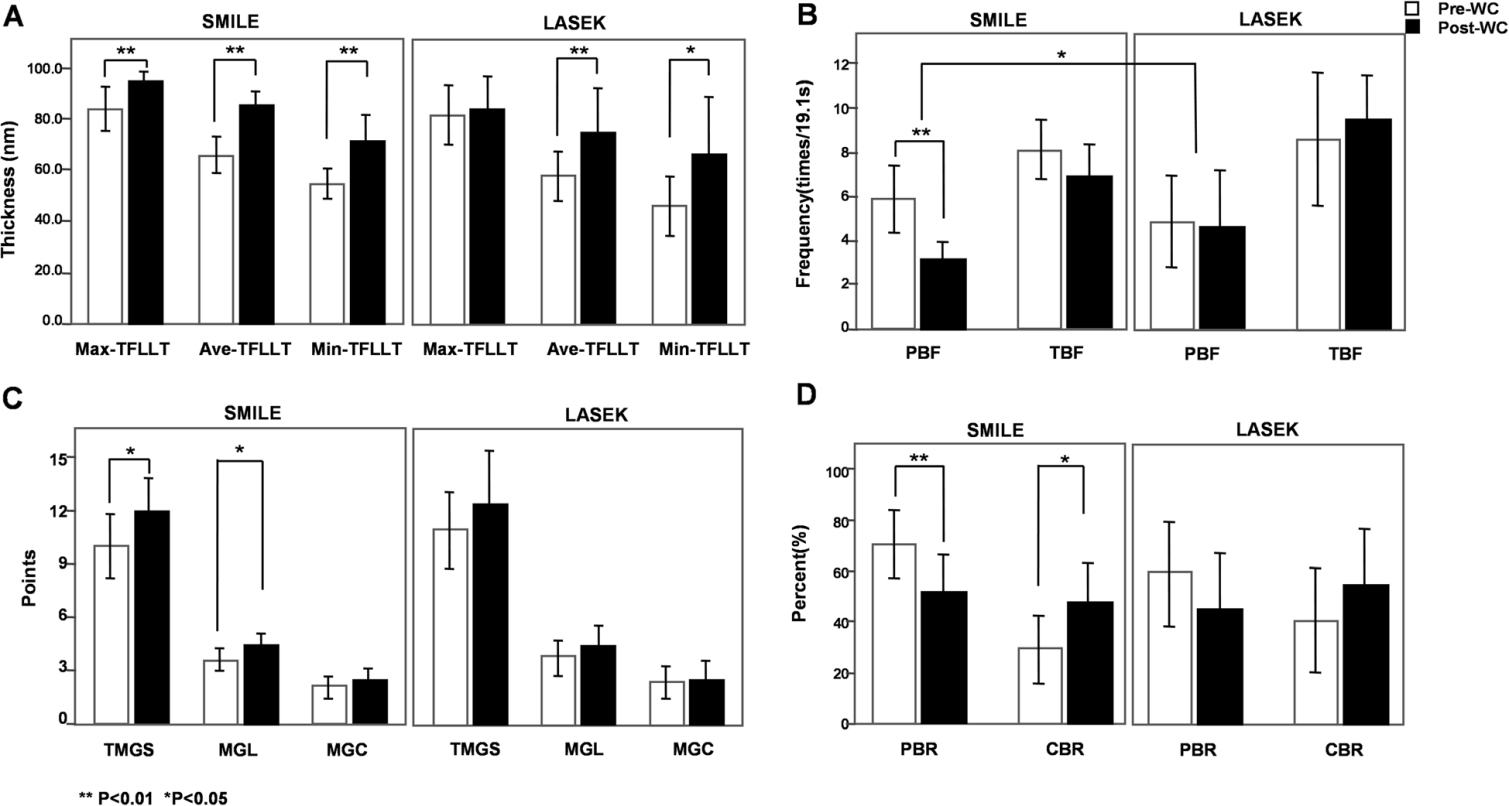
Change in TFLLT, blink frequency, meibomian glands scores and blink rate after WC following SMILE and LASEK. (**A**: Min-, Max- and Ave-TFLLT; **B**: total and partial blink frequency; **C**: different meibomian glands scores; **D**: complete and partial blink rate)

The mean values of Min-, Max- and Ave-TFLLT increased significantly after WC (*p* < 0.001, *p* = 0.023, *p* < 0.001, respectively, Fig. 1A; Table 1), whereas TFLLT-Std did not change. Figure 2 shows the contrast image under the LipiView display window before and after WC. Significant increases of Ave-, Max- and Min-TFLLT were observed in postoperative eyes in the SMILE group (*p* < 0.001, *p* = 0.006, *p* < 0.001, respectively), but only Ave- and Min-TFLLT increased significantly following WC in the LASEK group (*p* = 0.006 and 0.015, respectively). No significant difference was observed between surgeries (Fig. 3A, Table 2)

**Table 1:** Overall differences of indicators from before to after warm compress (WC)

	Pre-WC	Post-WC	*P* value
Lipid Layer			
TFLLT-Ave(nm)	63.08±17.37	81.89±19.30	0.000*
TFLLT-Max(nm)	82.86±19.62	91.00±13.81	0.023*
TFLLT-Min(nm)	51.70±16.28	70.43±26.60	0.000*
TFLLT-Std	5.38±2.60	7.84±9.28	0.109
TBUT(s)	5.38±2.19	6.38±2.16	0.000*
Meibomian Gland			
TMGS (0 to 45 points)	10.28±4.05	12.06±4.53	0.002*
MGL (0 to 15, n)	3.64±1.51	4.39±1.71	0.004*
MGC (0 to 15, n)	2.17±1.48	2.50±1.58	0.139
Blink pattern Parameters			
TBF (times/19.1s)	8.27±3.81	7.70±3.69	0.505
PBF (times/19.1s)	5.54±3.66	3.57±2.92	0.002*
CBR (%)	32.76±32.89	50.28±35.51	0.002*
PBR (%)	67.23±32.89	49.72±35.51	0.002*
LogMAR UDVA	-0.05±0.10	-0.08±0.09	0.270
LogMAR CDVA	-0.12±0.07	-0.13±0.07	0.317
SE (Diopter, D)	-0.47±0.52	-0.34±0.42	0.042*
K1	38.55±2.19	38.59±2.22	0.953
K2	39.44±2.12	39.45±2.19	0.899
Km	38.99±2.15	39.02±2.19	0.816
CCT (um)	462.47±44.03	471.57±49.01	0.006*
Total HOAs	0.63±0.51	0.85±0.38	0.593
Z (4, 0)	0.30±0.27	0.39±0.23	0.970
Z (3, 1)	-0.13±0.34	-0.15±0.34	0.101
Z (3, -1)	-0.43±0.46	-0.42±0.44	0.688

All data are expressed as the mean ± standard deviations

*TFLLT* tear film lipid layer thickness, *TBUT* tear film break-up time, *TMGS* total Meibomian gland secretion score, *MGL* glands secreting any liquid, *MGC* glands secreting clear liquid, *TBF* total blink frequency, *PBF* partial blink frequency, *PBR* partial blink rate, *CBR* complete blink rate, *UDVA* uncorrected distance visual acuity, *CDVA* corrected distance visual acuity, *SE* spherical equivalent, *CCT* central corneal thickness, *HOAs* higher order aberrations

*Significant difference

**Table 2:** Comparison between SMILE and LASEK before and after WC

	SMILE		*P* value	LASEK		*P* value	*P* value^#^
	Pre-WC	Post-WC		Pre-WC	Post-WC		
TFLLT-Ave(nm)	65.68 ± 18.22	85.00 ± 14.67	0.000^*^	57.67 ± 14.71	75.42 ± 26.10	0.006^*^	0.794
TFLLT-Max(nm)	83.60 ± 20.46	94.16 ± 9.58	0.006^*^	81.33 ± 18.53	84.42 ± 18.81	0.694	0.666
TFLLT-Min(nm)	54.48 ± 14.80	72.00 ± 22.70	0.000^*^	45.92 ± 18.31	67.17 ± 34.27	0.015^*^	0.642
TFLLT-Std	5.56 ± 2.87	6.36 ± 3.68	0.390	5.00 ± 1.95	10.92 ± 15.38	0.127	0.902
TBUT(s)	5.65 ± 2.12	6.26 ± 1.51	0.009^*^	4.82 ± 2.32	6.64 ± 3.20	0.008^*^	0.772
TMGS (0 to 45 points)	10.00 ± 4.39	11.92 ± 4.67	0.011^*^	10.91 ± 3.24	12.36 ± 4.39	0.102	0.700
MGL (0 to 15, n)	3.60 ± 1.55	4.40 ± 1.76	0.014^*^	3.72 ± 1.49	4.36 ± 1.69	0.142	0.710
MGC (0 to 15, n)	2.08 ± 1.55	2.52 ± 1.56	0.078	2.36 ± 1.36	2.45 ± 1.63	0.873	0.636
TBF (times/19.1s)	8.12 ± 3.35	6.84 ± 3.70	0.083	8.58 ± 4.78	9.50 ± 3.06	0.231	0.063
PBF (times/19.1s)	5.88 ± 3.87	3.08 ± 2.08	0.000^*^	4.83 ± 3.24	4.58 ± 4.10	0.754	0.030^*^
CBR (%)	29.08 ± 33.07	48.08 ± 36.30	0.012^*^	40.43 ± 32.54	54.84 ± 34.90	0.145	0.799
PBR (%)	70.91 ± 33.07	51.93 ± 36.30	0.008^*^	59.56 ± 32.54	45.14 ± 34.90	0.145	0.691
LogMAR UDVA	-0.05 ± 0.10	-0.07 ± 0.09	0.556	-0.05 ± 0.14	-0.10 ± 0.09	0.180	0.514
LogMAR CDVA	-0.12 ± 0.06	-0.13 ± 0.07	0.317	-0.13 ± 0.08	-0.13 ± 0.08	1.000	0.733
SE (Diopter, D)	-0.43 ± 0.45	-0.33 ± 0.41	0.200	-0.56 ± 0.66	-0.35 ± 0.49	0.068	0.857
K1	38.09 ± 2.04	38.08 ± 1.96	0.560	39.63 ± 2.28	39.79 ± 2.45	0.491	0.263
K2	39.01 ± 2.04	38.96 ± 1.99	0.348	40.44 ± 2.06	40.58 ± 2.33	0.541	0.486
Km	38.56 ± 2.04	38.52 ± 1.95	0.313	40.01 ± 2.17	40.19 ± 2.37	0.064	0.032^*^
CCT (um)	452.71 ± 35.09	458.38 ± 37.04	0.072	485.22 ± 55.83	502.33 ± 61.28	0.021^*^	0.125
Total HOAs	0.73 ± 0.52	0.89 ± 0.42	0.274	0.43 ± 0.41	0.73 ± 0.21	0.735	0.228
Z (4,0)	0.30 ± 0.26	0.37 ± 0.21	0.510	0.28 ± 0.31	0.46 ± 0.28	0.612	0.604
Z (3,1)	-0.11 ± 0.38	-0.14 ± 0.39	0.129	-0.16 ± 0.22	-0.21 ± 0.12	0.436	0.708
Z (3, -1)	-0.57 ± 0.42	-0.56 ± 0.38	0.901	-0.01 ± 0.32	0.02 ± 0.29	0.528	0.670

All data are expressed as the mean ± standard deviations

*TFLLT* tear film lipid layer thickness, *TBUT* tear film break-up time, *TMGS* total Meibomian gland secretion score, *MGL* glands secreting any liquid, *MGC* glands secreting clear liquid, *TBF* total blink frequency, *PBF* partial blink frequency, *PBR* partial blink rate, *CBR* complete blink rate, *UDVA* uncorrected distance visual acuity, *CDVA* corrected distance visual acuity, *SE* spherical equivalent, *CCT* central corneal thickness, *HOAs* higher order aberrations

*Significant difference (*P* < 0.05)

^#^Comparison between SMILE and LASEK

.

### Blink Pattern

Both PBF and PBR decreased significantly after WC (*p* = 0.002 in both cases, Fig. 1B, D; Table 1). Following SMILE, significant decreases were observed in PBF (*p* < 0.001) and PBR (*p* = 0.008), whereas an increase was observed in CBR (*p* = 0.012). There was no significant change in the LASEK subgroup. Except for PBF (*p* = 0.030), there was no significant difference between surgeries. (Fig. 3B, D; Table 2)

### TBUT and Meibomian Gland Function

The mean TBUT value increased significantly after WC (*p* < 0.001, Table 1). The differences were significant in both SMILE (*p* = 0.009) and LASEK (*p* = 0.008) subgroups, but not between surgeries (Table 2). There were statistically significant increases in TMGS and MGL (*p* = 0.002, 0.004, respectively), but not in MGC (Fig. 1C, Table 1). Significant increase of TMGS (*p* = 0.011) and MGL (*p* = 0.014) was observed in SMILE subgroup, whereas not in LASEK subgroup or between surgeries (Fig. 3C, Table 2).

### Safety and other clinical outcomes

No adverse events were observed during the study. After WC, the mean SE value and CCT increased significantly (*p* = 0.042, 0.006, respectively), while UDVA, CDVA, K_1_, K_2_, K_m_, total higher order aberrations (HOAs), Z (4, 0), Z (3, 1) and Z (3, -1) remained unchanged (*p* > 0.05) (Table 1). The increased CCT was significant in eyes following LASEK (*P* = 0.021), but not in eyes following SMILE (*p* = 0.072). The difference of K_m_ was only significant between surgeries (*p* = 0.032) but not within any subgroup. Beyond that, no significant differences of other parameters were observed within either subgroup or between surgeries. (Table 2)

### Correlation analysis

**Fig. 4:**
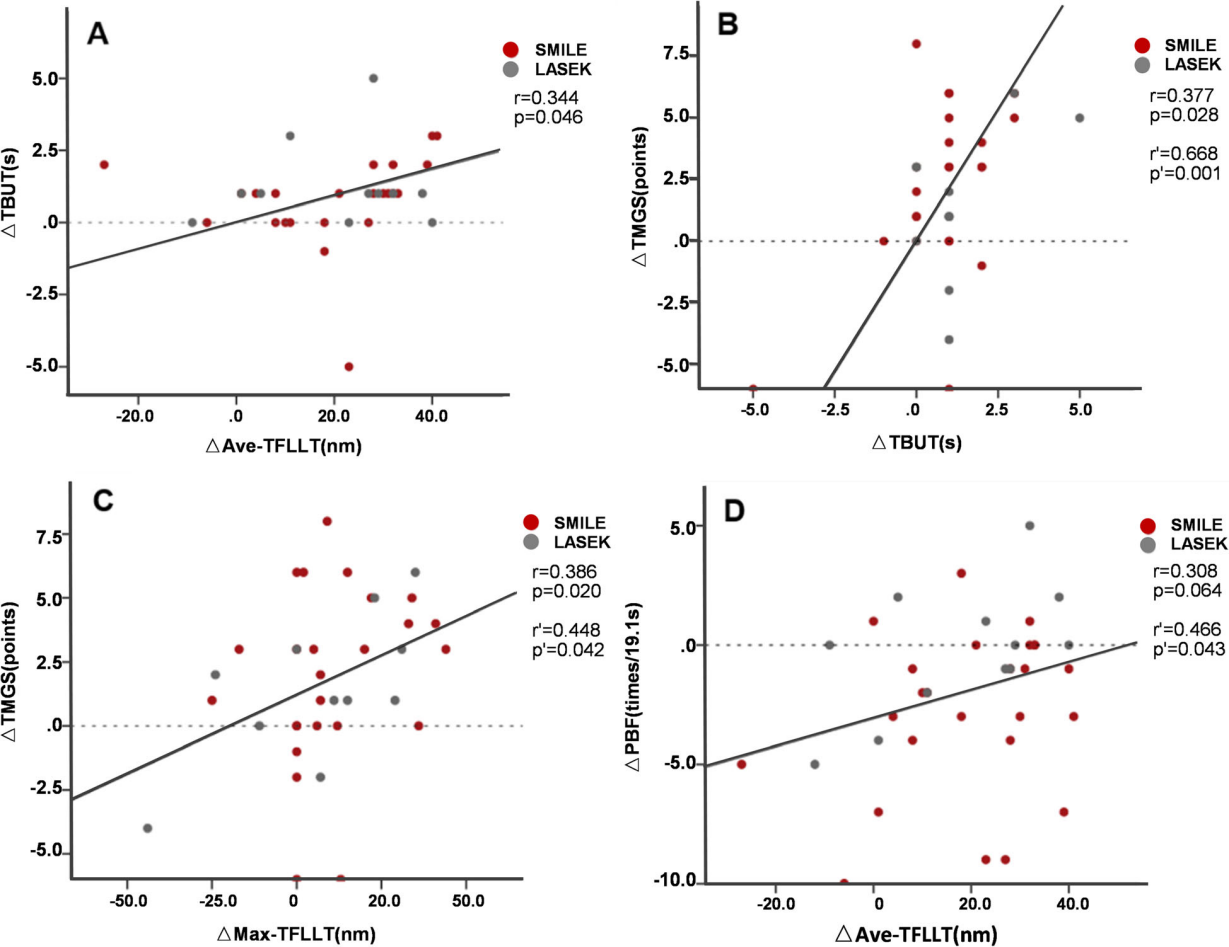
Correlations between (**A**) change in Ave-TFLLT and change in tear film break-up time (TBUT); **B** change in TBUT and change in total Meibomian gland secretion score (TGMS); **C** change in Max-TFLLT and change in TGMS; **D** change in Ave-TFLLT and change in partial blink frequency (PBF). R or *P*-value with superscript indicates result after adjustment for age, sex, and pre-operative SE

The change in TBUT (△TBUT) was positively correlated with △Ave-TFLLT (Fig. 4A) and △TGMS (Fig. 4B). △Max-TFLLT was positively correlated with △TMGS (Fig. 4C). After adjustment for age, sex and pre-operative SE, △TBUT was still positively correlated with △TMGS (Fig. 4B), also with △MGL (*r* = 0.649, *p* = 0.001), △MGC (*r* = 0.604, *p* = 0.004) and △TBF (*r* = 0.487, *p* = 0.025). △Max-TFLLT was still positively correlated with △TMGS (Fig. 4C), and △Ave-TFLLT was positively correlated with △PBF (Fig. 4D).

## Discussion

Dry eye syndrome is one of the most common postoperative complications of corneal refractive surgery (CRS), usually presenting as a transitory phenomenon during the early stages of recovery that naturally resolves several months later. However, complaints of persistent dry eye symptoms cannot be ignored in clinical practice. CRS was reported to be a risk factor for dry eye [13], which in turn increased the risk of refractive regression postoperatively [14]. It is therefore very important to improve the postoperative prognosis and break this vicious cycle by relieving chronic dry eye after CRS. According to previous report, CRS may adversely affect functional meibomian glands, which contributed to chronic tear film dysfunction after CRS [15]. Thermal treatments and eyelid massage are conventional therapy for patients with MGD, but when combined with WC, massage could induce rubbing-related deformation. The risk may be higher for cornea with refractive surgery, which may potentially increase risk of corneal ectasia [16]. So only WC was performed in this study under safety concern. Despite the proven efficacy of WC in various studies, poor compliance can’t be ignored due to time and temperature required [17]. Eyelid thermal pulsation (LipiFlow) therapy was previously reported to improve recalcitrant dry eye syndrome after CRS [18], but it also produced the highest per-treatment cost and was only recommended as second line therapy to MGD patients who were unresponsive to WC [19]. Portable spontaneous heating eye mask has a significant advantage of cost and accessibility over the LipiFlow, and simplify the application of heat therapy, which may improve compliance and make it convenient to use at-home.

Among the different tear layers, the outer lipid layer is thought to retard evaporation and increase stability of the tear film. Changes in distribution and decreased thickness of this layer can lead to dry eye [20], and TFLLT is significantly correlated with other dry eye indicators [21]. As a newly invented ocular surface interferometer, LipiView permits dynamic measurement of the lipid layer thickness with simultaneous recording of the blink pattern. The efficacy and repeatability of this instrument has been previously confirmed [22, 23], and it has been used in the evaluation of dry eye [24], MGD [25] and post-cataract surgery [26]. The repeatability and accuracy of TFLLT measurements with LipiView are superior to another commonly used instrument (Keeler Tearscope-Plus™) [23].

This study showed significant increases in TFLLT and TBUT after WC, and some improvements in Meibomian gland function. One possible explanation is that WC contributes to dilating the Meibomian gland ducts, melting meibum and promoting liquid secretion and thus further reducing the evaporation of tears, and increasing the stability of the tear film. This assumption agree with previous study, which reported that elevated eyelid temperature could unblock the meibomian glands, deliver more meibomian oil and ameliorating dry eye symptoms [27]. Jung et al. reported worse Meibomian gland structure and function in CRS patients following surgery relative to healthy individuals [15]. This being so, it is reasonable to assume that WC will confer greater benefits on postoperative patients than on healthy individuals, a possibility that warrants further investigation. Besides, we observed significant increases in TMGS and MGL but not in MGC, suggesting that the improvement occurred mainly in dysfunctional Meibomian glands by reducing obstruction rather than stimulating secretion. It’s worth noting that the partial recovery time for meibomian gland yielding clear liquid secretion was approximately 2 h in healthy eye [28]. In that case, the nonsignificant change of MGC may be partly related to unrecovered gland function. The recovery time of dysfunctional Meibomian glands was still unclear. Therefore, adequate interval time from baseline evaluation should be preserved in future studies to eliminate this impact.

The increased Ave-TFLLT and Min-TFLLT values were both higher than the upper limits of the deviations for previously repeated measurements (from − 9 to 16 nm) [22], indicating a significant change. The smaller variations of Max-TFLLT relative to Ave-TFLLT and Min-TFLLT may result from limitations of the measurement capability of the former by the LipiView detection range (up to 100 nm), particularly in patients with high Max-TFLLT baseline values. It is worth noting that the dry eye was more prevalent in Asian populations than Australian populations [29, 30]. Moreover, the risk of chronic dry eye after refractive surgery was also significantly higher among Asians [31], suggesting the possibility of ethnic differences in dry eye. Mean Ave-TFLLT was recently measured at 54 nm among Asians [22], which seems significantly lower than previous report in dry eye patients (76 ± 25 nm) [32]. Given that Meibomian gland disease was also more common among patients with low TFLLT (≤ 60 nm) [32], clinicians attending Asian patients should be aware of the risks of post-operative dry eye problems.

Blinking is important for promoting the secretion of lacrimal gland, recoating the cornea with tears, and maintaining good visual acuity. Decreased blinking frequency is an important factor contributing to postoperative tear film instability [31, 33], but the effectiveness of blinking is often overlooked in clinical practice. In our study, both PBF and PBR decreased significantly after WC, indicating improved blink efficiency. TRF appeared unaffected both by WC and by other variables that we measured. It is reasonable to assume that relative to total blinking, partial blinking might have a bigger impact on long-term tear film stability. This conjecture was supported by a previous study, which indicated that extent of blinking rather than total blink rate was the determinant for tear film instability [34]. A possible explanation is that incomplete blinking may cause defects in re-distribution of the mucin and lipid layers, thereby increasing tear evaporation and decreasing lipid layer thickness. Moreover, the impaired corneal innervation and reduced protective blink reflex following CRS may increase partial blinking rate and tear film instability [35]. It is particularly noteworthy that the blinking habit is trainable, and blink efficiency exercises can achieve long-lasting benefits [35, 36]. Methods to modify blinking habits and reduce partial blinking are therefore worth exploring for postoperative patients with chronic dry eye.

Association between tear film, meibomian gland function and blinking pattern has been noticed in many clinical studies [24, 33]. To explore the role of TFLLT, blink pattern and meibomian gland function in tear film stability after CRS, we conducted multiple correlation analyses. We show that variation in TBUT was positively correlated with that of TFLLT and Meibomian gland scores, and the latter two parameters were also positively correlated. These results implied that TFLLT and Meibomian gland function may be a protective factors of tear film stability after CRS. Besides, TFLLT measurement was reported to be affected by demographic factors such as age and sex [24]. After adjustment, the variation in PBF was positively correlated with Ave-TFLLT, suggesting that blinking pattern may also be involved in the increase of TFLLT after WC. This is not surprising as the tear film lipid layer is originated from meibum, secreted from the meibomian gland and spread onto the ocular surface with each blink [37]. TFLLT, Meibomian gland score and blink pattern might also be effective indices for tear film evaluation, like the traditional indicator TBUT.

To further compare benefits between the two surgery types, we analyzed the effects of WC on SMILE and LASEK patients. There was no significant difference between two groups before WC. After WC, TFLLT-Max, CBR and Meibomian gland function improved significantly in the SMILE group, whereas PBF and PBR decreased. No significant changes of any above indicators were observed in the LASEK group, indicating that different surgical procedures may differ in their impact on the recoverability of TFLLT and blink pattern. Previous study reported that the Meibomian gland parameters showed worsening after refractive surgery, more in the SMILE group than LASEK group [15], which agrees with the baseline Meibomian gland evaluation result in our study. In that case, WC may be more beneficial for the SMILE group. Moreover, although SMILE and LASEK are both flapless corneal refractive surgeries with only superficial corneal ablation, the former had worse recovery of corneal sensitivity following surgery, besides, association among the blinking rate, TFLLT, and corneal sensitivity was also noticed [38]. Therefore, the varying degree of postoperative corneal sensitivity recovery may partly explain the different change of TFLLT and blink pattern following SMILE and LASEK. However, at more than 2 years after surgery, we observed no significant difference between surgeries except PBF. Similarly, Chung B, et al. also reported that the decrease in corneal sensitivity recovered to baseline level at 6 months after both SMILE and LASEK [38]. Accordingly, we presumed that the long-term difference between SMILE and LASEK might be slight, which need to be further investigated.

To further assess the safety and other clinical outcomes of this treatment, we measured visual acuity, SE, keratometry, corneal thickness, and aberration but observed no significant differences before and after WC except CCT. According to Niimi J, et al., increased tear osmolarities are associated with thinner corneas, and eye closure can create a hypoosmotic environment by reducing tear evaporation and tear drainage, and lead to increase of CCT [39]. This may partly explain the increase of CCT during eye-closed WC procedure. Besides, the tear evaporation can be further reduced with increased TFLLT during WC, which may also contribute to low tear osmolarity established within a short time. Moreover, thicker cornea was associated with slower deswelling [39], which may be involved in the difference between surgeries, considering the baseline CCT value seems higher in LASEK group. No significant change of keratometry was observed after WC, which indicated this WC method would not cause obvious corneal deformation in CRS patients. The Solomon et al. reported blurred vision and decreased visual acuity after WC [40]. Interestingly, the slight but significant decrease we observed in SE was the opposite of that. Different WC methods and measurement sequences among studies may account for this discrepancy. For example, vision measured immediately after WC might be blurred. At present study, WC went smoothly without adverse effects or complaints of discomfort, demonstrating that short-term WC was safe for post-CRS patients.

There are some limitations to our study. First, both eyes were treated simultaneously and there was a lack of control group. As mentioned above, eye closure itself can influence tear evaporation and drainage, in addition, diurnal variations were detected in both tear flow and corneal thickness [41, 42]. To eliminate bias, nonheated eye mask with similar material could have been applied to the contralateral eye as a control. Second, this study focused exclusively on changes shortly after WC. According to the Tear Film and Ocular Surface Society’s Dry Eye Workshop II (TFOS DEWS II), the TBUT improvement following a single application of WC can last for up to 30 min and repeated eyelid warming achieve a stable improvement on both tear film and meibomian gland function [17]. Studies over a longer time scale and regular WC sessions are needed as a follow-up. Third, the TFOS DEWS II also recommended optimal WC treatment temperature of ≥ 40℃, which refers to temperature on palpebral conjunctiva and the gland, not on the external skin of the eyelids [17]. Immediately after removal of a heated eyebag, the maximal internal eyelid temperature was about 1.5℃ less warm than the mean surface temperature of the eyebag [43]. Considering the mean temperature was 40.7 °C in this study, the actual temperature on palpebral conjunctiva and the gland may be a little inadequate. An infrared thermometer along with the WC device in recommended, for even temperature maintenance and accurate temperature record. Fourth, LipiView only measured the lower part of the cornea and we therefore lacked information about overall corneal condition. This may have increased the likelihood that our analyses exaggerated the role of incomplete blinking on tear thinning, because the consequences of such blinking are localized on the inferior cornea [36]. Finally, the sample size in this study is small, and a larger sample size is expected for further investigations.

## Conclusions

In conclusion, our study demonstrated that WC may temporally increase TFLLT and TBUT, decrease incomplete blink and partly improve Meibomian gland function in dry eye patients after SMILE and LASEK. It seems more beneficial for the former. Further investigation of long-term clinical efficacy and safety needs to be performed.

## Data Availability

Data and materials are available upon request from the corresponding author at doctzhouxingtao@163.com.

## Abbreviations

WC: Warm compress
SMILE: Femtosecond laser small incision lenticule extraction
LASEK: Laser-assisted subepithelial keratomileusis
LASIK: Laser-assisted in situ keratomileusis
FS-LASIK: Femtosecond laser-assisted in situ keratomileusis
TBUT: Tear film break-up time
TFLLT: Tear film lipid layer thickness
MGS: Meibomian secretory function scores
Min-: Minimum
Max-: Maximum
Ave-: Average
TMGS: Total Meibomian secretory function scores
MGL: Number of glands secreting any liquid
TBF: Total blink frequency
MGC: Number of glands secreting clear liquid
CBR: Complete blink rate
PBF: Partial blink frequency
PBR: Partial blink rate
CCT: Central corneal thickness
UDVA: Uncorrected distance visual acuity
CDVA: Corrected distance visual acuity
SE: Spherical equivalent
ICUs: Interferometric color units
std: Standard deviation
HOAs: Higher order aberrations
CRS: Corneal refractive surgery
